# Radiation-pressure-induced surface deformation of transparent liquids due to laser beams under oblique incidence

**DOI:** 10.1016/j.pacs.2025.100781

**Published:** 2025-11-10

**Authors:** B. Anghinoni, L.C. Malacarne, C. Jacinto, M.L. Baesso, B. Lendl, N.G.C. Astrath

**Affiliations:** a Department of Physics, Universidade Estadual de Maringá, Maringá, 87020-900, PR, Brazil; b Nano-Photonics and Imaging Group, Instituto de Física, Universidade Federal de Alagoas, Maceió, 57072-900, AL, Brazil; c Institute of Chemical Technologies and Analytics, Technische Universität Wien, Vienna, 1060, Austria

**Keywords:** Radiation pressure, Surface deformation, Green’s function, Photo-induced mirror, Optomechanics

## Abstract

We consider the deformation of transparent liquid surfaces under the influence of radiation pressure generated from a gaussian laser beam with oblique incidence. It is seen that the beam intensity cross-section at the surface becomes elliptical, presenting a distinct behavior from the models with cylindrical symmetry previously adopted in the literature to describe experimental observations. Semi-analytical solutions to the equilibrium deformations in terms of Green’s functions are also presented. Based on these solutions, numerical simulations for an air–water interface were carried out, showing that in typical scenarios the modified beam intensity generates larger deformations as function of the incidence angle, with differences up to order of 10 nm, which should be observable by current experimental optical techniques. A proposal of such measurement employing a photo-induced mirror technique was also simulated, where it was seen that the expected experimental signal does present the sensitivity required to distinguish between the considered models.

## 1 Introduction

Opto-mechanical effects such as radiation-induced deformations are important to many modern technologies, especially for applications in optical trapping and manipulation of elastic matter [1], [2], [3], [4], [5]. In fact, the deformation of liquids due to optical forces was first observed more than five decades ago [6] – however, due to the nano-scale displacements involved only in recent years quantitative works have been reported [7], [8], [9], [10], [11]. Although first attributed to momentum transfer from light, this deformation phenomenon stems from optically-induced stresses which change the local mechanical pressure within the medium [12], [13], [14], [15]. Indeed, characterizing the momentum transfer by light and its mechanical effects have proven to be very challenging topics, both in theory and experiment, being fundamentally tied to the centenary and still ongoing Abraham-Minkowski controversy [13], [16], [17], [18], [19].

A full description of the radiation-induced deformations as function of time is generally a very difficult topic if the sample being illuminated is smaller than the beam radius – a very common situation in nanoscale optical manipulations techniques [20], [21]. This occurs mainly because the deformed sample’s surface dynamically affects the radiation pressure by locally changing the excitation beam’s incidence angle. Nonetheless, the opto-hydrodynamics of micro-droplets and micro-bubbles has been studied semi-analytically [22], [23] in linear deformation regime. On the other hand, for large deformations and complex geometries one must resort to computational techniques [24], [25].

The situation is usually much more amenable when studying the deformation of free fluid surfaces whose lateral dimensions are much greater than the incident beam radius. Typically, the radiation-pressure-induced surface deformations in air–liquid interfaces due to gaussian beams are expansions of order 10 nm, quickly decaying over a lateral length of a few unities of beam radius, which on its turn is very frequently in the range of 10–100μm [7], [8], [9], [10], [26]. Therefore, when calculating the radiation pressure acting on flat interfaces, the angle of incidence can be considered unperturbed by the sample, being consequently constant in time. This greatly simplifies the theoretical opto-hydrodynamic analysis.

The dynamical deformation of liquid surfaces due to the action of gaussian beams was first theoretically addressed, to our knowledge, in Ref. [27] by Lai and Young, where the capillary–gravity waves generated by the normal incidence of a gaussian beam were described (see Appendix A). Hereafter, the steady-state surface deformation taken from Ref. [27] for a cw beam will be referred to as YL solution. Many works in the specialized literature subsequently employed the YL solution to study the equilibrium deformations occurring under non-normal laser incidence [9], [10], [28], [29], [30], [31]. Although in general a decent agreement with the experiments has been reported, it must be noticed that in the absence of the cylindrical symmetry inherent to normally-incident gaussian beams the YL solution is no longer valid, requiring a generalization for the radiation pressure as a function of the beam’s incidence angle relative to the surface. This generalization and the associated surface deformations will be developed in this work, where a semi-analytical solution in terms of Green’s functions will be presented. Notice that the full description of the transmission and reflection of gaussian beams at oblique incidence on dielectric slabs has been presented in earlier work [32], but our focus here is distinct as it lies in the radiation pressure acting on the dielectric flat interface and the deformations induced by it.

## 2 Gaussian beam intensity at flat interfaces under oblique incidence

**Fig. 1:**
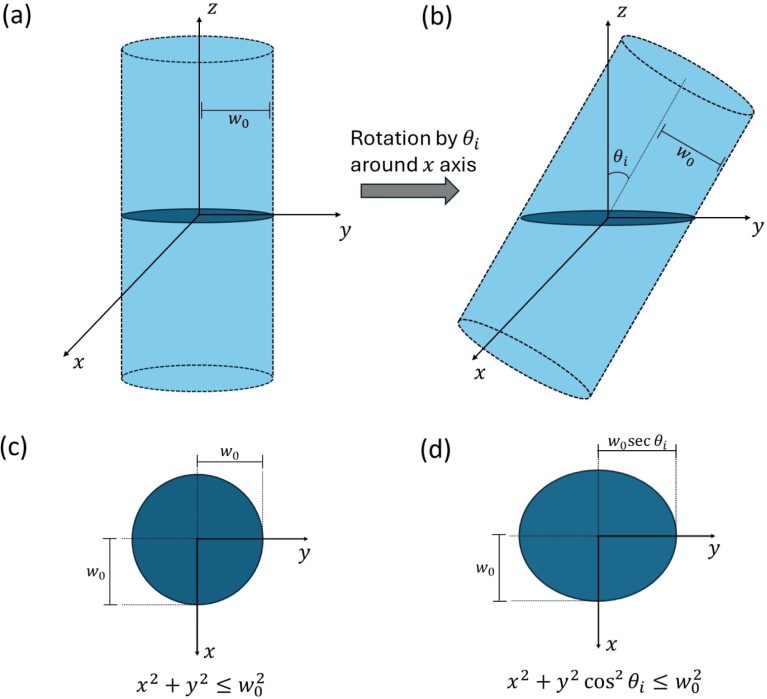
(a) and (b) Rotation of the beam intensity profile by an angle $\theta_{i}$ around the x axis. The shaded regions depict the beam intensity cross-section at the interface plane z=0, which changes from a circle to an ellipse under oblique incidence, seen in (c) and (d). In the figure, $\theta_{i}=30{}^{\circ}$, which generates a semi-major axis approximately 15% larger than the semi-minor axis.

Consider the propagation of an arbitrary laser beam inciding on an interface between two dielectric media. Under oblique incidences, the cross-section of the laser beam intensity at the interface is generally different from its cross-section at normal incidence. Specifically, for gaussian beams in the fundamental mode inciding on flat interfaces, the intensity cross-section changes from a circle to an ellipse. To show this, we consider here a collimated gaussian beam of radius $w_{0}$ propagating in −z direction inciding on an interface located at the z=0 plane with incidence angle $\theta_{i}$ ($0\leq \theta_{i}<90{}^{\circ}$). We first consider this beam at normal incidence to obtain an expression for the beam intensity cross-section at the interface. By adopting the beam intensity spatial extent to be one unity of $w_{0}$, it is easy to see that the aforementioned cross-section is given by a circle, described by the set of points satisfying the equation $x^{2}+y^{2}\leq w_{0}^{2}$, with z=0. Correspondingly, the associated beam intensity is proportional to $\;e^{-\left(x^{2}+y^{2}\right)/w_{0}^{2}}$, regardless of the beam’s polarization. The expression for the oblique-incidence beam intensity profile, on its turn, is obtained by first actively rotating, by an angle $\theta_{i}$ around the x axis, the cylinder defined from the circular cross-section expanded for arbitrary z (shown in Fig. 1(a)), then subsequently calculating the intersection of this rotated cylinder with the z=0 plane, as depicted in Fig. 1(b). Mathematically, the coordinates of the rotated cylinder, (X,Y,Z), are given in terms of the initial cylinder coordinates (x,y,z) as

(1) $$\left[\begin{array}{c} X\\ Y\\ Z \end{array}\right]=\left[\begin{array}{ccc} 1 & 0 & 0\\ 0 & \cos\theta_{i} & -\sin\theta_{i}\\ 0 & \sin\theta_{i} & \cos\theta_{i} \end{array}\right]\left[\begin{array}{c} x\\ y\\ z \end{array}\right].$$

Solving the linear system from Eq. (1) yields X=x and $Y=y\cos\theta_{i}-z\sin\theta_{i}$, while Z is unimportant here. As we are interested in the beam cross-section profile at the interface, we set z=0, which gives $Y=y\cos\theta_{i}$. Thus, the beam cross-section changes from the circle $x^{2}+y^{2}\leq w_{0}^{2}$ (seen in Fig. 1(c)) to an ellipse described by the equation $x^{2}+y^{2}\cos^{2}\theta_{i}\leq w_{0}^{2}$ (seen in Fig. 1(d)). From the ellipse equation in reduced form, ${\left(x/w_{0}\right)}^{2}+{\left(y/w_{0}\sec\theta_{i}\right)}^{2}\leq 1$, we see that the semi-minor axis of the ellipse is $w_{0}$ while the semi-major axis is $w_{0}\sec\theta_{i}$. As the secant is a monotonically increasing function in the domain $0\leq \theta_{i}<90{}^{\circ}$, the beam profile becomes elongated in the y axis as $\theta_{i}$ increases, as expected. Accordingly, the beam intensity at the interface changes its gaussian spatial dependence to $\;e^{-\left(x^{2}+y^{2}\cos^{2}\theta_{i}\right)/w_{0}^{2}}$, where the loss of cylindrical symmetry can be easily noticed for $\theta_{i}\neq 0$. This intensity dependence is valid for any beam polarization.

## 3 Semi-analytical solutions to surface deformations

Under external fields, a free surface of a liquid medium will deform due to the action of existent surface and body forces. In equilibrium, with no velocity field present within the liquid, viscosity effects can be neglected, and the Navier–Stokes equation reduces to a Laplace equation as [33] $\nabla^{2}P=\nabla \cdot f_{b}$, where P is the mechanical pressure in the liquid and $f_{b}$ is the resultant body force density acting on it. Laplace’s equation is well-known to possess unique solutions and, in this situation, the surface deformation for transparent liquids can be shown to occur exclusively due to the electro- and magnetostriction force densities [12], [14], [34]. Additionally, the radiation pressure acting on the surface is uniquely related to these striction forces and corresponds exactly to the well-known Abraham-Minkowski force density [13], [14]. The above considerations allow us to describe the surface deformation of the liquid at equilibrium in terms of the Young–Laplace law, which relates the pressure jump at the interface (ΔP) and the deformed surface geometry as $\Delta P=-\gamma \nabla \cdot \overset{ˆ}{n}$, where γ is the surface tension parameter and $\overset{ˆ}{n}$ is the normal unit vector, given as $\overset{ˆ}{n}=\left(\partial_{x}h,\partial_{y}h,-1\right)/{\left[1+{\left(\partial_{x}h\right)}^{2}+{\left(\partial_{y}h\right)}^{2}\right]}^{1/2}$, with h(x,y) denoting the magnitude of the surface deformation relative to the interface plane z=0. For small deformations, the denominator of the expression for $\overset{ˆ}{n}$ can be taken as unity, and we obtain $\nabla \cdot \overset{ˆ}{n}\approx \nabla^{2}h\left(x,y\right)$, where $\nabla^{2}$ is the Laplacian operator. Therefore, the Young–Laplace law in linear deformation regime yields [33]

(2) $$\gamma \nabla^{2}h\left(x,y\right)-\left(\rho_{2}-\rho_{1}\right)gh\left(x,y\right)+P_{\mathrm{rad}}\left(x,y\right)=0,$$

where $\rho_{1}$ and $\rho_{2}$ are the mass densities of the media (assuming beam propagation occurs from medium 1 to medium 2), g is the local gravity acceleration and $P_{\mathrm{rad}}\left(x,y\right)$ is the radiation pressure generated by the fields. For vanishing deformation at x,y→±∞, Eq. (2) admits solutions in the form $h\left(x,y\right)=\int \;\int G\left(x,y;x^{\prime },y^{\prime }\right)P_{\mathrm{rad}}\left(x^{\prime },y^{\prime }\right)\;dx^{\prime }\;dy^{\prime }$, where $G\left(x,y;x^{\prime },y^{\prime }\right)$ is the Green’s function of the problem, given as (see Appendix B for the calculation)

(3) $$G\left(x,y;x^{\prime },y^{\prime }\right)=\frac{1}{2\pi \gamma }K_{0}\left(a{\left[{\left(x-x^{\prime }\right)}^{2}+{\left(y-y^{\prime }\right)}^{2}\right]}^{1/2}\right).$$

Here, $K_{0}$ denotes the 0th order modified Bessel function of the second kind and $a=\sqrt{\gamma^{-1}\left(\rho_{2}-\rho_{1}\right)g}$. To obtain h(x,y), we now need to characterize the radiation pressure $P_{\mathrm{rad}}\left(x,y\right)$.

### 3.1 Radiation pressure

The radiation pressure for non-magnetic, linear and transparent dielectric interfaces is [13], [14] $P_{\mathrm{rad}}=\left({ɛ}_{2}-{ɛ}_{1}\right){\left|E\right|}_{\mathrm{avg}}^{2}/2$, where ${ɛ}_{2}$ and ${ɛ}_{1}$ denote the electric permittivity of the media and ${\left|E\right|}_{\mathrm{avg}}^{2}$ is the spatial average (across the interface) of the squared modulus of the electric field. The coordinate system shown in Fig. 1 was adopted, where medium 1 occupies the region z>0 and medium 2 the region z<0. According to the boundary conditions stemming from Maxwell’s equations, the longitudinal field component $E_{z}$ is not continuous across the interface. Therefore, choosing a procedure to calculate its spatial average is necessary. As shown in Ref. [14], in the so-called microscopic Ampère (MA) formulation a simple arithmetic mean is adopted. In other earlier works from literature, both theoretical and experimental, a different averaging procedure was employed [9], [10], [28], [29], [31], [35], [36], [37], based on the continuity of the longitudinal component of the electric displacement vector field, $D_{z}$, at the interface. For linear media, this condition is mathematically equivalent to averaging the product $ɛE_{z}$. However, as extensively discussed by Barnett and Loudon in Ref. [38], the permittivity ɛ is a quantity obtained from a macroscopic averaging over a single material and therefore is ill-defined at surfaces – i.e., $E_{z}$ can be averaged at surfaces, as done in the MA formulation, but ɛ cannot. Incidentally, proceeding with this physically unjustified assumption yields a radiation pressure that can be shown to correspond to a geometric mean of $E_{z}$ [14], [37]. Thus, the numerical values obtained for typical dielectric interfaces are close in magnitude for both averaging methods (especially for small values of $\theta_{i}$), partially justifying the agreement found in experimental works [9], [10], [28], [29], [31], [35]. Such difference in radiation pressure, however, should be distinguishable in realistic setups, as discussed in the next section.

As $P_{\mathrm{rad}}$ is proportional to the beam intensity at the surface, we may write $P_{\mathrm{rad}}\left(x,y\right)=f\left(\theta_{i}\right)\;e^{-\left(x^{2}+y^{2}\cos^{2}\theta_{i}\right)/w_{0}^{2}}$, where $f\left(\theta_{i}\right)$ contains the dependence on $\theta_{i}$ from the model adopted for the radiation pressure and also the polarization. Following the discussion from Section 1, in the system under study the angle of incidence is not appreciably affected by the surface deformation. Thus, for the specific purpose of calculating radiation pressure acting on flat interfaces due to gaussian beams, the incident beam’s wavefront can, to good approximation, be considered locally plane, with a single incidence angle $\theta_{i}$. This allows us to describe the transmission and reflection phenomena in terms of Fresnel coefficients $t_{p}\left(\theta_{i}\right)$, $t_{s}\left(\theta_{i}\right)$ and $r_{p}\left(\theta_{i}\right)$ as [14]

(4) $$f_{\mathrm{MA}}^{\left(p\right)}\left(\theta_{i}\right)=\;A\;\left[t_{p}^{2}\left(\theta_{i}\right)\;\cos^{2}\theta_{t}\;+\;\frac{{\left(1\;+\;r_{p}\left(\theta_{i}\right)\right)}^{2}\sin^{2}\theta_{i}}{2}+\frac{t_{p}^{2}\left(\theta_{i}\right)\sin^{2}\theta_{t}}{2}\right]\;,$$

(5) $$f_{\mathrm{lit}}^{\left(p\right)}\left(\theta_{i}\right)=A\left[\left(\sin^{2}\theta_{i}+\cos^{2}\theta_{t}\right)t_{p}^{2}\left(\theta_{i}\right)\right]$$

and

(6) $$f^{\left(s\right)}\left(\theta_{i}\right)=At_{s}^{2}\left(\theta_{i}\right),$$

where $A=\left[\left({ɛ}_{r2}-{ɛ}_{r1}\right)P_{0}\right]/\left(\pi n_{1}cw_{0}^{2}\right)$, with $P_{0}$ denoting the cw beam power, ${ɛ}_{r1}$ and ${ɛ}_{r2}$ the relative electric permittivities, and $\theta_{t}=\sin^{-1}\left(n_{1}\sin\theta_{i}/n_{2}\right)$ is the transmitted angle at the interface, with the refractive indices $n_{1}=\sqrt{{ɛ}_{r1}}$ and $n_{2}=\sqrt{{ɛ}_{r2}}$. Besides, $f_{\mathrm{MA}}^{\left(p\right)}$ is associated with the MA formulation and p polarization (where the arithmetic spatial average is employed), $f_{\mathrm{lit}}^{\left(p\right)}$ with the literature version and p polarization (where the geometrical spatial average is employed) and $f^{\left(s\right)}$ with the s polarization, which is the same in both approaches as no averaging across the interface is necessary in this case. In the Fresnel coefficients, r denotes reflection and t transmission, with the subscripts indicating the polarization.

The surface deformation h(x,y) is therefore given semi-analytically as

(7) $$h\left(x,y\right)=\frac{f\left(\theta_{i}\right)}{2\pi \gamma }\;\;\int \;\;\;\int \;\;K_{0}\left(a{\left[{\left(x-x^{\prime }\right)}^{2}+{\left(y-y^{\prime }\right)}^{2}\right]}^{1/2}\right)\times \;\exp\;\left[-\left(x^{\prime 2}\;+\;y^{\prime 2}\;\cos^{2}\;\theta_{i}\right)/w_{0}^{2}\right]\;\;\;dx^{\prime }\;\;dy^{\prime }\;,$$

where the double integration is to be performed over the $x^{\prime }-y^{\prime }$ plane.

## 4 Numerical results and discussion

To calculate the surface deformation numerically, we adopt throughout this section the realistic values $w_{0}=50\;\;\mu m$ and cw beam power of 5 W. We also consider an air-to-water beam incidence, with [39] ${ɛ}_{r1}=1.0003$, ${ɛ}_{r2}=1.7689$, $\rho_{1}=1.2\;\mathrm{kg}/m^{3}$, $\rho_{2}=998.2\;\mathrm{kg}/m^{3}$, γ=72.8 mN/m and $g=9.79\;m/s^{2}$.

**Fig. 2:**
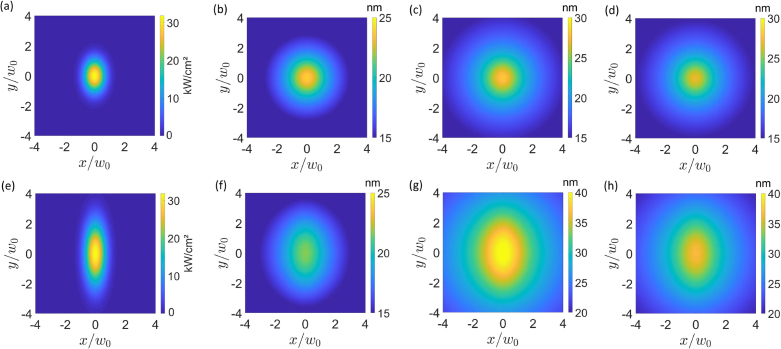
(a) and (e) Beam intensity for $\theta_{i}=30{}^{\circ}$ and $\theta_{i}=70{}^{\circ}$, respectively. (b)–(d) Surface deformations h(x,y) for $\theta_{i}=30{}^{\circ}$ with s polarization, with p polarization in MA model, and with p polarization in literature model, respectively. (f)–(h) Same as before, but for $\theta_{i}=70{}^{\circ}$.

In Fig. 2 we see the two-dimensional beam intensity cross-section (left column) and surface deformations h(x,y) (remaining columns) for angles of incidence $\theta_{i}=30{}^{\circ}$ and $\theta_{i}=70{}^{\circ}$ with both polarizations and both models for p-polarized radiation pressure. The results show strictly positive values of the order 10 nm for the deformations, indicating a water surface expansion, in accordance with the earlier literature with the same media and similar beam parameters [7], [8], [9], [10]. When $\theta_{i}=30{}^{\circ}$, the semi-major axis is only about 15% larger than the semi-minor axis, and correspondingly the elliptical profile is barely noticeable in both the intensity profile and the associated surface deformations. For $\theta_{i}=70{}^{\circ}$, on the other hand, the semi-major axis is almost 3 times larger than the semi-minor axis, generating a very pronounced elliptic profile for the intensity. The surface deformations also follow this tendency. We notice that the s-polarized deformations (Figs. 2(b) and 2(f)) depend weakly on the incidence angle while the p-polarized deformations (Figs. 2(c), 2(d), 2(g) and 2(h)), on their turn, present peaks that increase significantly with $\theta_{i}$. This fact is expected as the p-polarized beam has an extra non-negligible field component acting on the interface ($E_{z}$) when compared to the s polarization.

**Fig. 3:**
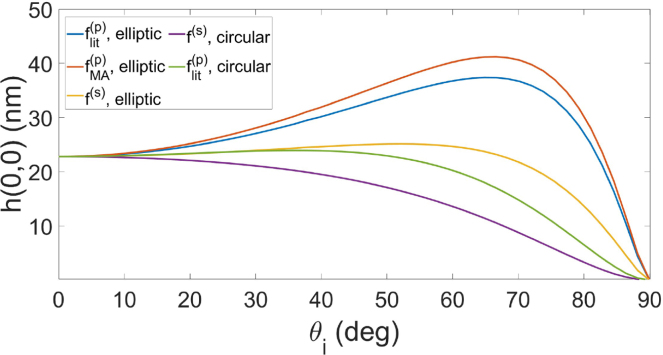
Surface deformations at beam center for air-to-water propagation as function of angle of incidence according to the considered models with both polarizations. The curves with circular profile depend on $\theta_{i}$ only through the Fresnel coefficients, while the curves with elliptic profile depend on $\theta_{i}$ through both beam intensity and Fresnel coefficients.

Fig. 3 shows the deformations at the beam center, h(0,0), as function of the incidence angle $\theta_{i}$. The distinct $f\left(\theta_{i}\right)$ from Eqs. (4) to (6) were considered, along with the circular and elliptic beam intensity cross-sections. Notice that the circular intensity profile, which was adopted in the YL solution, is independent of $\theta_{i}$. The deformations in this case (purple and green lines) are dependent on $\theta_{i}$ through the Fresnel coefficients only, while for the elliptic profile derived in this work (yellow, blue and red lines) such dependence is due to both the beam intensity and the Fresnel coefficients. We can see in Fig. 3 that all cases produce an expansion of the water surface, since all the $f\left(\theta_{i}\right)$ functions are strictly positive for ${ɛ}_{2}>{ɛ}_{1}$. Besides, the elliptic and circular profiles produce very similar deformations, for both polarizations, if the angle is smaller than 15°. As this angle increases, however, relatively larger deformations are obtained from the elliptic profile, with a maximum difference around 66°. More specifically, these differences reach approximate values of $\Delta h^{\left(s\right)}=10\;\mathrm{nm}$ and $\Delta h^{\left(p\right)}=20\;\mathrm{nm}$ for s and p polarizations, respectively, where the MA formulation was adopted to define $\Delta h^{\left(p\right)}$. Additionally, for elliptical profile and p polarization, the maximum difference in deformation between the two models described (blue and red lines in Fig. 3) is $\Delta h_{\mathrm{rad}}\approx 4\;\mathrm{nm}$. In principle, such values can be measured by current optical techniques such as photo-induced mirror and interferometry [7], [40], [41].

From the discussion presented in Ref. [14], it is expected that the averaging method employed in MA formulation provides better agreement with experiments. This was indeed confirmed in a recent work where the deformation of a free surface of water under oblique laser incidence was measured with a sub-nanometer-precise interferometric technique [42]. However, notice that in this reference the elliptic profile of the beam cross-section at the interface was argued to be unimportant in interpreting the observations. This claim is initially in discordance with the results presented in this work, both on physical and numerical grounds. Another important experimental work was reported in Ref. [10], where a millimetric water droplet under oblique incidence was examined. With values for $w_{0}$ and $P_{0}$ similar to the ones adopted in our simulations, the observed deformations matched the circular-profile model for $\theta_{i}$ up to 55°, with a sub-5 nm precision. The maximum differences between these observations and the results predicted by our model are about 10 nm (not shown here), which are too small to support definitive conclusions within the given precision. Thus, more detailed investigations are currently necessary to clarify the aforementioned discrepancies.

The distinct behavior of the surface deformations in Fig. 3 arises from the interplay between the angular dependence of the radiation-pressure functions and the beam cross-section profile. For s polarization, the deformation decreases monotonically with incidence angle, while for p polarization the coexistence of parallel and normal field components yields a maximum region due to the product of decreasing transmission/reflection coefficients with increasing sine terms. The elliptic beam profile further enhances this effect: because the gaussian factor in the double integral shown in Eq. (7) broadens along the $y^{\prime }$ axis as $\theta_{i}$ increases (proportional to $\sec\theta_{i}$, as discussed in Section 3), the integrated contribution grows with angle, shifting and amplifying the deformation maximum toward larger $\theta_{i}$. This explains why the elliptic case shows stronger and higher-angle peaks, including the large deformation near 66° for p polarization.

**Fig. 4:**
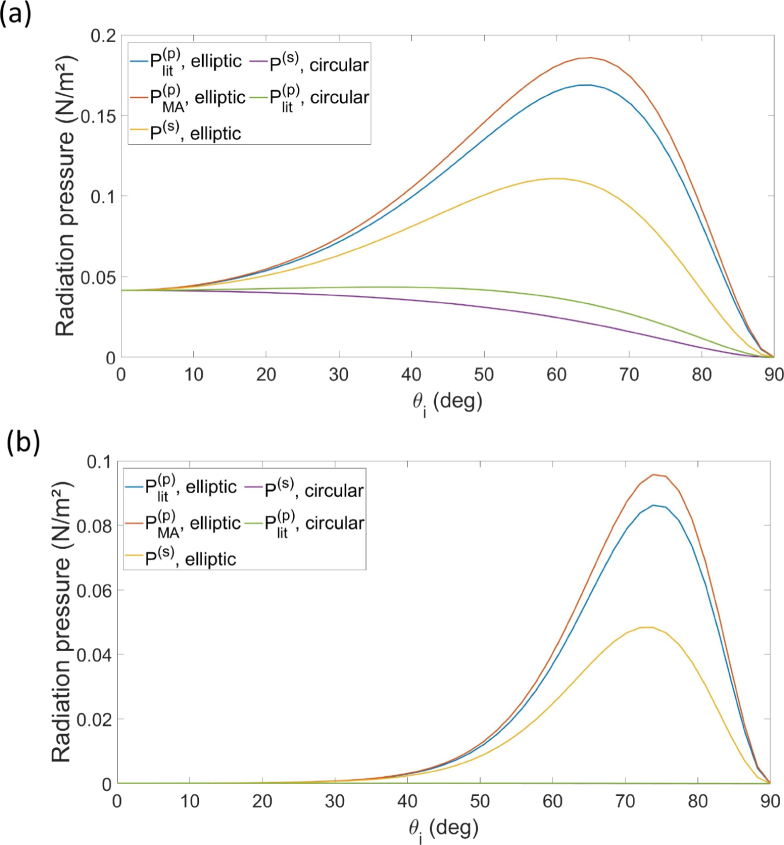
Radiation pressure as function of $\theta_{i}$ according to the considered models and both polarizations, calculated at (a) $\left(x,y\right)=\left(0,w_{0}\right)$ and (b) $\left(x,y\right)=\left(0,2w_{0}\right)$. In (b), the lines related to the radiation pressure with circular profile are negligible.

Notice that as $P_{\mathrm{rad}}\left(x,y\right)=f\left(\theta_{i}\right)\;e^{-\left(x^{2}+y^{2}\cos^{2}\theta_{i}\right)/w_{0}^{2}}$, the spatial dependence of the radiation pressure is entirely given by the beam intensity. If we then calculate $P_{\mathrm{rad}}$ at the origin, its value will be the same for both circular and elliptic cross-sections, regardless of the incidence angle $\theta_{i}$ – although it will still be different for s and p polarizations, as already explored in earlier literature [35]. In fact, in our system the behavior of $P_{\mathrm{rad}}$ as function of $\theta_{i}$ is the same for circular and elliptic intensities for the whole (x,0) line, as the rotation of the beam incidence only affects the intensity in y-direction (see Fig. 1). Therefore, the effects of the different intensity cross-sections in the radiation pressure must be observed at points outside the (x,0) line. Such effects can be clearly seen in Fig. 4, where we have the radiation pressure calculated, as function of $\theta_{i}$, at points $\left(x,y\right)=\left(0,w_{0}\right)$ (Fig. 4(a)) and $\left(x,y\right)=\left(0,2w_{0}\right)$ (Fig. 4(b)) for the different models considered and both polarizations (the notation follows that of Fig. 3 for the f functions). Accounting for the correct intensity profile leads to higher values of radiation pressure for both polarizations. In particular, in Fig. 4(b) we see that the curves associated with the circular profile model are negligible (purple and green lines coincide), while the elliptic profile still present non-negligible radiation pressure.

To facilitate the experimental distinction between the circular and elliptic models for the beam intensity cross-section (and also for the p-polarized radiation pressure), it is convenient to consider transparent dielectric media that present overall larger deformations. In systems where the surface deformation is induced by radiation pressure, the effects of the surface tension typically dominate over the effects of gravity. This can be readily quantified through the dimensionless Bond number, Bo, which is a measure of the relative magnitude between buoyancy and capillary effects [24], and is given here as $\mathrm{Bo}=w_{0}^{2}a^{2}$. In our simulated setup, Bo is of order 10^−4^ – thus, the surface tension dictates the magnitude of the deformation, with lower γ values providing larger deformations. This conclusion holds even if we use $w_{0}\sec\theta_{i}$ instead of $w_{0}$ to define the characteristic length (unless $\theta_{i}$ is extremely close to 90°, which is a very unpractical situation). In this context, water is not a particularly interesting choice for experimental studies as γ is known to be large — nevertheless, it was employed in the numerical simulations presented here due to its frequent appearance in the related experimental works. Additionally, higher values of ${ɛ}_{r2}$ increase the radiation pressure, which should also produce larger deformations.

It is important to clarify the limits of validity of the small-deformation approximation used in our analysis. Formally, the condition was specified in Section 4 as ${\left(\partial_{x}h\right)}^{2}+{\left(\partial_{y}h\right)}^{2}\ll 1$. A more intuitive criterion is to consider the ratio between the maximum deformation amplitude and the incident beam radius, which directly estimates the characteristic surface slope. Small-deformation conditions then correspond to slope values much smaller than unity. For the 10 nm-order deformations shown in Fig. 2 with beam radius $w_{0}=50\;\mu m$, the corresponding slope is $∼10^{-3}$, which satisfies our condition. Considering the beam radius constant with order 10μm, deformations one order of magnitude larger (possible in samples with higher permittivity and lower surface tension, as discussed in the last paragraph) would still yield slopes much smaller than unit, and thus remain within the applicability of the small-deformation approximation.

The consideration of beams with arbitrary modulation would naturally introduce a time dependence in the surface deformation itself so the Young–Laplace law in Eq. (2) cannot be applied anymore, being replaced by the capillary–gravity wave equation [27]. In this situation, semi-analytical solutions in terms of Green’s functions as developed here become much more complicated to obtain and are left as a topic for future works. In any case, the effects of the presented elliptic beam intensity cross-section should not be altered.

### 4.1 Photo-induced mirror technique simulations

**Fig. 5:**
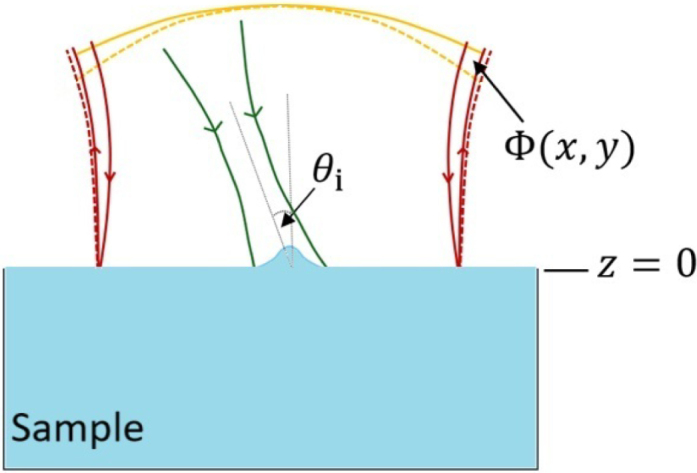
Principle of operation of the photo-induced mirror technique. Green arrows represent the pump beam inciding with angle $\theta_{i}$ (reflection and transmission are not shown). Red arrows depict the probe beam, whose reflected components are seen in solid lines for the flat interface and dashed lines for the deformed surface. Correspondingly, the solid and dashed yellow lines are the wavefront of the initial and distorted reflected probe beams, respectively, which are related by the phase difference Φ(x,y).

The so-called photo-induced mirror (PIM) is an all-optical pump-probe experimental technique able to observe surface deformations of nanometric order in condensed matter samples [7], [40]. The distortion of a probe beam wavefront due to the alterations in the sample’s surface, illustrated in Fig. 5, is monitored in the far-field region by a photodetector. From the variation in the intensity measured at the center of this photodetector, it is possible to obtain information on many parameters from the sample. The PIM technique has been successfully employed in earlier literature to analyze and measure the surface deformation of low-absorbing samples under normal laser incidence [7], [8], [11], [43]. It is a particularly convenient technique to study the two-dimensional surface deformations reported here as the setup is sensitive to the whole surface morphology, and not only to the magnitude of the peak deformation.

In the present case with $\theta_{i}\neq 0$, the excitation beam and therefore the surface deformation do not present cylindrical symmetry. The observed intensity in equilibrium (relative to the unperturbed case), denoted by S, can be generalized as

(8) $$S=|\int_{-\infty }^{+\infty }\int_{-\infty }^{+\infty }\exp\left[\;-\;\frac{i\pi }{\lambda_{p}}\;\left(\frac{x^{2}\;+\;y^{2}}{z_{2}}\;+\;\frac{x^{2}\;+\;y^{2}}{R_{1p}}\right)+\frac{x^{2}+y^{2}}{w_{1p}^{2}}-i\Phi \left(x,y\right)\right]\;\;dx\;d{y|}^{2}\;,$$

where $i=\sqrt{-1}$, $\Phi \left(x,y\right)=4\pi h\left(x,y\right)/\lambda_{p}$ is the phase difference, with $\lambda_{p}$ denoting the probe beam’s wavelength, $w_{1p}$ the probe beam’s minimum radius, $R_{1p}$ the probe beam’s wavefront curvature at the point of minimum radius and $z_{2}$ the distance between the sample surface and the photodetector. The theoretical deformations h(x,y) described in this work can therefore be inserted into Eq. (8) and compared to the experimental value measured for S as a function of $\theta_{i}$. This approach allows us to verify the validity of the radiation pressure under oblique incidence presented here, as well as the best model for the radiation pressure of p-polarized beams.

**Fig. 6:**
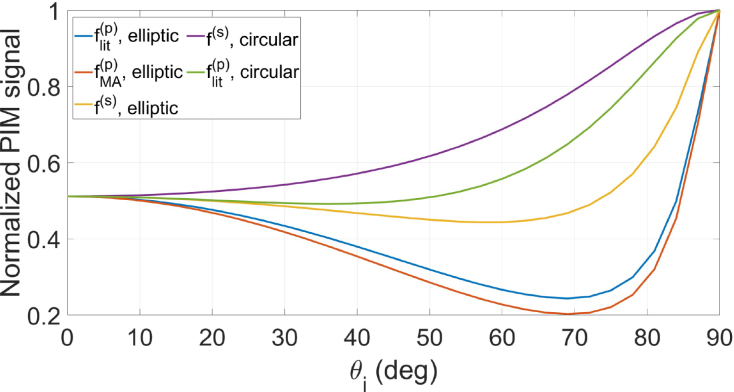
Normalized PIM signal S for a water sample in steady-state considering the different models and polarizations.

Fig. 6 shows the simulated normalized PIM signal S as function of $\theta_{i}$ for water under steady-state conditions, i.e., after the surface deformation is fully built due to the cw laser excitation. As in Fig. 3, the two polarizations and the two models are considered. The additional setup parameters were taken from Ref. [8] as follows: $\lambda_{p}=632\;\mathrm{nm}$, $w_{1p}=$ 3.64 mm, $R_{1p}=33.2$ cm, $z_{2}=6.8$ m. We can see that the signal in this configuration behaves similarly to the peak deformations, i.e., distinction between the models is clearer in the range of $50{}^{\circ}<\theta_{i}<80{}^{\circ}$, approximately. Typically, the uncertainties in a PIM setup are about 0.2% (see Refs. [7], [8] for example), which are undoubtedly precise enough to discriminate the curves shown in Fig. 6 in the range just mentioned. Thus, the PIM technique corresponds to a robust experimental possibility to test the models presented in this work.

Although no experimental measurements under oblique incidence are presented here, the simulations provided in this section aim to demonstrate the feasibility of using the PIM technique for testing our theoretical predictions and to guide future experimental implementations. Identifying the best model for the optically-induced radiation pressure under general beam incidence is important for modern opto-mechanical and optical trapping applications, while also improving our knowledge on optical forces in matter in the context of the long-standing Abraham-Minkowski controversy.

## 5 Conclusions

In summary, we obtained semi-analytical solutions for the deformation of flat interfaces of transparent dielectric liquids due to the radiation pressure generated from a gaussian beam under oblique incidence. In simulations for an air–water interface with realistic experimental parameters, such deformations were seen to be larger when compared to the earlier literature semi-analytical model, which adopted a circular profile for the beam intensity cross-section at oblique incidences. The largest differences in deformation were approximately 10 nm and 20 nm for s and p polarizations, respectively. Two distinct models for the radiation pressure for p polarization were also considered, and the expected difference in deformations is about 4 nm. All these differences should be possible to be observed with modern optical techniques and, moreover, they can be made larger if necessary by choosing transparent liquids with lower surface tension and/or higher refractive index. Specifically, we simulated the expected signal from a photo-induced mirror pump-probe setup, which was seen to present the necessary sensitivity to measure the water surface deformations and identify the best models precisely.

The theory developed here further elucidates the interaction of light with dielectric interfaces, contributing to the resolution of the ongoing Abraham-Minkowski controversy and also to opto-mechanical and optical manipulation applications.

## CRediT authorship contribution statement

**B. Anghinoni:** Writing – review & editing, Writing – original draft, Investigation, Formal analysis, Conceptualization. **L.C. Malacarne:** Investigation, Formal analysis. **C. Jacinto:** Investigation, Formal analysis. **M.L. Baesso:** Investigation, Formal analysis. **B. Lendl:** Investigation, Formal analysis. **N.G.C. Astrath:** Writing – review & editing, Writing – original draft, Supervision, Resources, Project administration, Investigation, Funding acquisition, Formal analysis, Conceptualization.

## Declaration of competing interest

The authors declare the following financial interests/personal relationships which may be considered as potential competing interests: N.G.C. Astrath reports financial support was provided by State University of Maringá.

## Data Availability

Data will be made available on request.

## Appendix A Semi-analytical solution for the cylindrical-symmetric case

For convenience, we show here a short derivation of the result for surface deformation under normal incidence and cw regime from Ref. [27]. If the beam intensity cross-section is circular, the radiation pressure at the interface depends only on the cylindrical coordinate r, and therefore Eq. (2) is reduced to

(9) $$\gamma \nabla_{r}^{2}h\left(r\right)-\left(\rho_{2}-\rho_{1}\right)gh\left(r\right)+f\left(\theta_{i}\right)\;e^{-2r^{2}/w_{0f}^{2}}=0,$$

where $w_{0f}$ is the radius defined for the field amplitude, related to the radius of the beam intensity as $w_{0f}^{2}/2=w_{0}^{2}$.

Now, we apply the Hankel transform (also known as Fourier–Bessel transform) to both sides of Eq. (9). This generates $\nabla_{r}^{2}\to -k^{2}$ and $\;e^{-2r^{2}/w_{0f}^{2}}\to \left(w_{0f}^{2}/4\right)\;e^{-k^{2}w_{0f}^{2}/8}$, from where we get

(10) $$\overset{\sim }{h}\left(k\right)=\frac{w_{0f}^{2}f\left(\theta_{i}\right)}{4\gamma }\frac{\;e^{-k^{2}w_{0f}^{2}/8}}{k^{2}+a^{2}},$$

where $\overset{\sim }{h}\left(k\right)$ is the Hankel transform of h(r) and $a^{2}=\left(\rho_{2}-\rho_{1}\right)g/\gamma$. The solution is then obtained by performing the inverse Hankel transform, which yields

(11) $$h\left(r\right)=\frac{w_{0f}^{2}f\left(\theta_{i}\right)}{4\gamma }\int_{0}^{\infty }\frac{\;e^{-k^{2}w_{0f}^{2}/8}}{k^{2}+a^{2}}J_{0}\left(kr\right)k\;dk,$$

where $J_{0}$ is the 0th order Bessel function of the first kind. This is the semi-analytical solution for the equilibrium surface deformation given in Ref. [27].

## Appendix B Semi-analytical solution for the non-cylindrical-symmetric case

The differential equation given in Eq. (2) is a case of the non-homogeneous Helmholtz equation, which is known as the screened Poisson equation. The oblique incidence breaks the cylindrical symmetry of the radiation pressure profile, so the solution from Ref. [27] adopted in earlier literature, given in Appendix A, cannot be applied. In seeking analytical or semi-analytical solutions, one traditional way is to find the Green’s function for the problem. In our case, the Green’s function $G\left(x,y;x^{\prime },y^{\prime }\right)$ must satisfy the differential equation $\gamma \nabla^{2}G-\left(\rho_{2}-\rho_{1}\right)gG=-\delta \left(x-x^{\prime }\right)\delta \left(y-y^{\prime }\right)$, where δ(x) denotes the Dirac delta distribution. Besides, the function G must also satisfy the same boundary conditions pertaining to the deformation h(x,y). Such conditions for h are h(x→±∞,y)=h(x,y→±∞)=0 and are physically justified by the assumption that the typical lateral length of the interface is much larger than the beam radius. By applying the Fourier transform to the differential equation for G, we get

(12) $$G\left(k_{x},k_{y};x^{\prime },y^{\prime }\right)=\frac{1}{\gamma }\frac{\;e^{\;i\left(k_{x}x^{\prime }+k_{y}y^{\prime }\right)}}{k_{x}^{2}+k_{y}^{2}+a^{2}},$$

which is inverted as

(13) $$G\left(x,y;x^{\prime },y^{\prime }\right)\;=\;\frac{1}{4\pi^{2}\gamma }\;\int \;\int \;\frac{\;e^{-\;i\left[k_{x}\left(x-x^{\prime }\right)+k_{y}\left(y-y^{\prime }\right)\right]}}{k_{x}^{2}+k_{y}^{2}+a^{2}}\;\;dk_{x}\;\;dk_{y},$$

where the double integral is to be performed over the $k_{x}-k_{y}$ plane and $a^{2}=\gamma^{-1}\left(\rho_{2}-\rho_{1}\right)g$. Now, we transform the integral into polar coordinates in the k-space, $\left(k_{r}\cos\theta ,k_{r}\sin\theta \right)$, obtaining

(14) $$G\left(x,y;x^{\prime },y^{\prime }\right)=\frac{1}{4\pi^{2}\gamma }\int_{0}^{\infty }\int_{0}^{2\pi }\frac{\;e^{-\;ik_{r}\left|r-r^{\prime }\right|\cos\theta }}{k_{r}^{2}+a^{2}}k_{r}\;d\theta \;dk_{r},$$

where $\left|r-r^{\prime }\right|={\left[{\left(x-x^{\prime }\right)}^{2}+{\left(y-y^{\prime }\right)}^{2}\right]}^{1/2}$. By using the property [44] $\int_{0}^{2\pi }\cos\left(n\theta \right)\exp\left(ix\cos\left(\theta -\phi \right)\right)\;d\theta =2\pi i^{n}J_{n}\left(x\right)\cos\left(n\phi \right)$, where $J_{n}$ denotes the nth order Bessel function of the first kind, the integration in θ yields

(15) $$G\left(x,y;x^{\prime },y^{\prime }\right)=\frac{1}{2\pi \gamma }\int_{0}^{\infty }\frac{J_{0}\left(k_{r}\left|r-r^{\prime }\right|\right)}{k_{r}^{2}+a^{2}}k_{r}\;dk_{r}.$$

The integration in $k_{r}$ finally leads to [45]

(16) $$G\left(x,y;x^{\prime },y^{\prime }\right)=\frac{1}{2\pi \gamma }K_{0}\left(a{\left[{\left(x-x^{\prime }\right)}^{2}+{\left(y-y^{\prime }\right)}^{2}\right]}^{1/2}\right),$$

where $K_{0}$ is the 0th order modified Bessel function of the second kind. As $K_{0}\left(x\to \pm \infty ,y\right)=K_{0}\left(x,y\to \pm \infty \right)=0$, the Dirichlet boundary conditions are satisfied. Thus, the solutions we seek are $h\left(x,y\right)=\iint G\left(x,y;x^{\prime },y^{\prime }\right)P_{\mathrm{rad}}\left(x^{\prime },y^{\prime }\right)\;dx^{\prime }\;dy^{\prime }$, where the double integral must be calculated for the $x^{\prime }$-$y^{\prime }$ plane. It can be numerically verified that the solution obtained here coincides with the solution for the cylindrical-symmetric case if $\theta_{i}=0$, as expected.
